# Phase 1 cohort expansion study of LY3023414, a dual PI3K/mTOR inhibitor, in patients with advanced mesothelioma

**DOI:** 10.1007/s10637-021-01086-6

**Published:** 2021-03-04

**Authors:** Marjorie G. Zauderer, Evan W. Alley, Johanna Bendell, Enrica Capelletto, Todd M. Bauer, Sophie Callies, Anna M. Szpurka, Suhyun Kang, Melinda D. Willard, Volker Wacheck, Anna M. Varghese

**Affiliations:** 1 Memorial Sloan Kettering Cancer Center, New York, NY USA; 2 Present Address: Taiho Oncology Inc, Princeton, NJ USA; 3 Cleveland Clinic Florida, Weston, FL USA; 4 Sarah Cannon Research Institute / Tennessee Oncology, Nashville, TN USA; 5 University of Turin, Torino, TO Italy; 6 Eli Lilly and Company, Indianapolis, IN USA

**Keywords:** Mesothelioma, LY3023414, PI3K/mTOR inhibitor, Solid tumor

## Abstract

*BACKGROUND* LY3023414 is a selective, ATP competitive inhibitor of class I PI3K isoforms, mTORC1/2 and DNA-PK. A Phase 1 dose escalation, 200 mg twice daily (BID) of LY3023414 was the determined recommended phase 2 dose (RP2D). We report the antitumor activity and safety of LY3023414 monotherapy in patients with advanced mesothelioma.*METHODS* Patients enrolled had advanced malignant pleural or peritoneal mesothelioma with measurable disease, ECOG PS 0–1, were refractory or ineligible to receive standard therapies. Patients received LY3023414 200 mg BID. This dose expansion cohort is intended to evaluate preliminary antitumor activity of LY3023414 by overall response rate. Safety, tolerability and pharmacokinetics were assessed. Biomarkers associated with treatment response was an exploratory endpoint. *RESULTS* Forty-two patients received LY3023414 for a median duration of 11.2 weeks (range: 1.1–53.0). One patient had a confirmed partial response (PR) (ORR 2.4%). Three patients had an unconfirmed PR. Seventeen patients had stable disease (SD) (DCR 43%). Most common adverse events (AEs) included fatigue (43%), nausea (43%), decreased appetite (38%), vomiting (33%), and diarrhea (29%). AEs were mostly mild or moderate. Grade ≥ 3 AEs were reported for 21% of patients with fatigue as the most frequent event (10%). Alterations of *BAP1* were identified in 11/19 patients as the most common molecular aberration, followed by *SETD2* and *NF2* alterations. No obvious pattern of genetic changes/mutations in single genes or pathways was associated with anti-tumor activity. *CONCLUSION* LY3023414 monotherapy (200 mg BID) demonstrated an acceptable and manageable safety profile with limited single-agent activity in patients with advanced mesothelioma. **ClinicalTrials.gov identifier:** NCT01655225; **Date of registration:** 19 July 2012.

**Supplementary Information:**

The online version contains supplementary material available at 10.1007/s10637-021-01086-6.

## Introduction

The phosphatidylinositol 3-kinase/mammalian target of rapamycin (PI3K/mTOR) pathway is vital in regulating physiological processes such as cell growth and proliferation. In the development of malignant disease, its activation has been reported in >30% of various solid tumor types [1, 2]. Pharmacological inhibition of PI3K/mTOR blocks tumor growth and survival signaling in different tumor xenograft models [3]. Several dual PI3K/mTOR inhibitors are currently under investigation as monotherapy or in combination with standard of care therapies. Besides allosteric mTOR inhibitors (everolimus and temsirolimus), delta isoform specific PI3K inhibitors are currently approved for clinical use [4]. However, for solid tumors with a high incidence of aberrant PI3K pathway activation, PI3K/mTOR inhibitor monotherapy could be employed in larger patient populations [5, 6].

Mesothelioma is a rare cancer that arises from the mesothelial cells lining the chest, heart, abdomen, or testes. About 3000 new cases are diagnosed each year in the USA, further underlining an unmet need for the treatment of malignant mesothelioma [7, 8]. In particular, for recurrent disease, there are limited treatment options available [9, 10]. Malignant mesotheliomas are characterized by loss of phosphatase and tensin homologue on chromosome 10 (PTEN) and activation of PI3K signaling in up to 62% and 84% of all cases, respectively [11, 12]. PTEN loss was reported as a strong, independent negative prognostic biomarker for overall survival in patients with mesothelioma. This justifies the need to target the PI3K/mTOR pathway in patients diagnosed with malignant mesothelioma [12]. While unrelated to the PI3K/mTOR pathway, previous literature characterized *NF2* and *BAP1* as some of the most commonly mutated genes in patients diagnosed with mesothelioma [13]. Therefore, these genes could also be considered as further biomarkers for disease progression in this population.

LY3023414 (Eli Lilly and Company; Indianapolis, IN, USA) is an orally available and selective inhibitor of class I PI3K isoforms, mTORC1/2, and DNA-PK, with high solubility across a wide pH range. In nonclinical studies, LY3023414 has demonstrated potent in vivo target inhibition that was linked to anti-tumor efficacy [14].

This trial (NCT01655225) was a multi-cohort phase 1a study investigating the safety and tolerability and pharmacokinetics of LY3023414 in patients with advanced and/or metastatic cancer. Based on data from the phase 1a portion of the trial, the recommended phase 2 dose (RP2D) of LY3023414 monotherapy was established to be 200 mg twice daily (BID) [15]. This phase 1 expansion cohort evaluated the safety and efficacy (preliminary antitumor activity) of LY3023414 monotherapy in patients diagnosed with malignant mesothelioma.

## Materials and methods

### Study design and treatment

This phase 1 multicenter, nonrandomized, open-label study of LY3023414 consisted of 2 parts: Part A, dose escalation using a 3 + 3 design to identify the recommended phase 2 dose (RP2D) and Part B, for cohort expansions enrolling patients with advanced and/or metastatic tumors, including one cohort for mesothelioma (ClinicalTrials.gov identifier: NCT01655225). The primary objective of the expansion cohort was to evaluate antitumor activity of LY3023414. Secondary objectives were to determine the safety and toxicity profile and characterize pharmacokinetic (PK). Exploratory endpoint included biomarker assessments.

This study was conducted in accordance with the Consensus ethics principles derived from international ethics guidelines, including the Declaration of Helsinki and Council for International Organizations of Medical Sciences (CIOMS), International Ethical Guidelines, International Council for Harmonisation Guidelines for Good Clinical Practice (ICH GCP), and applicable local regulations. The protocol was approved by the ethics committees of all participating centers, and all patients provided written informed consent before study entry.

### Patient population

Patients eligible had advanced or metastatic malignant pleural or peritoneal mesothelioma of epithelioid, sarcomatoid, or mixed-type, and no previous PI3K/mTOR inhibitor therapy. Further inclusion criteria were an Eastern Cooperative Oncology Group performance status (ECOG PS) score of 0 or 1 and measurable disease per Response Evaluation Criteria in Solid Tumor (RECIST v1.1). Patient must have adequate organ function and baseline tumor tissue for biomarker analysis. Patients with serious preexisting medical conditions, symptomatic central nervous system metastasis, were excluded from study enrollment.

### Safety assessments

Adverse events (AEs) were graded by the National Cancer Institute’s (NCI) Common Terminology Criteria for Adverse Events (CTCAE) 4.0 and coded according to the Medical Dictionary for Regulatory Activities (MedDRA).

### Efficacy assessments

Tumor responses were evaluated by the investigators as per modified RECIST for mesothelioma at all even cycles through cycle 8, then every 2–4 cycles as clinically indicated by CT or MRI [16]. Disease control rate (DCR) was defined as (CR + PR + SD). Anti-tumor effect will be summarized by the overall response rate (ORR) defined as (CR + PR). Change in tumor size was derived for all patients on therapy with measurable disease at baseline and at least 1 post-treatment assessment.

### Pharmacokinetic analysis

The pharmacokinetic sampling schedule and the analysis method for LY3023414 were already disclosed as part of disclosure of the dose escalation phase data by Bendel et al. [15].

### Exploratory biomarker analysis

Tumor samples were collected for exploratory analysis of PI3K/mTOR pathway related biomarkers. Genetic alterations identified by prior locally performed testing (i.e., next generation sequencing tests performed on archival tissue) were collected as available and analyzed for association with clinical outcomes. Biomarkers were assessed for any associations with clinical outcomes.

## Results

### Patient characteristics

A total of 42 patients diagnosed with advanced mesothelioma were enrolled and received at least one dose of the study drug. Patients’ baseline characteristics are summarized in Table 1. The median age of patients treated was 69 years and the majority were male (74%), white (86%), with ECOG PS 1 (74%). Among the treated patients, 41 (98%) had prior systemic therapy, 41 (98%) had prior radiotherapy, and 33 (79%) had prior surgery. At baseline, 13 patients (31%) had a histological finding of epithelioid mesothelioma cells. Epithelioid was among the most frequently diagnosed histological subtype (Table 1).

**Table 1:** Patient demographics and baseline characteristics

Characteristic	*N* = 42
Age: median (yrs) (Range)	69 (52–81)
Race, n (%)	
White	36 (85.7)
Black or African American	0
Asian	2 (4.8)
Missing	4 (9.5)
Gender, n (%)	
Male	31 (73.8)
Female	11 (26.2)
ECOG PS, n (%)	
0	11 (26.2)
1	31 (73.8)
Prior anti-cancer therapies, n (%)	
Prior systemic therapy	41 (97.6)
Prior radiotherapy	41 (97.6)
Prior surgery	33 (78.6)
Baseline pathological diagnosis, n (%)	
Epithelioid	26 (61.9)
Sarcomatoid	2 (4.8)
Biphasic	2 (4.8)
Other	12 (29)

*ECOG PS* Eastern Cooperative Oncology Group, N total number of patients n number of patients in the specified category, *PS* performance status, *Yrs* years

### Treatment exposure

Median duration of LY3023414 study treatment was 11.2 (1.1–53.0) weeks with a median relative dose intensity of 86.1%. At least one dose adjustment was required in 18 patients (43%) enrolled. Dose reduction and dose interruption was reported in 14 (33%) and 8 (19%) patients, respectively. The most common reason for study discontinuation was progressive disease (48%). Further reasons for discontinuation are listed in (Supplementary Table 1). Discontinuation due to AEs was reported in three patients (7%), one patient each with dyspnea, fatigue, and general disorders, respectively.

### Antitumor activity

**Fig. 1:**
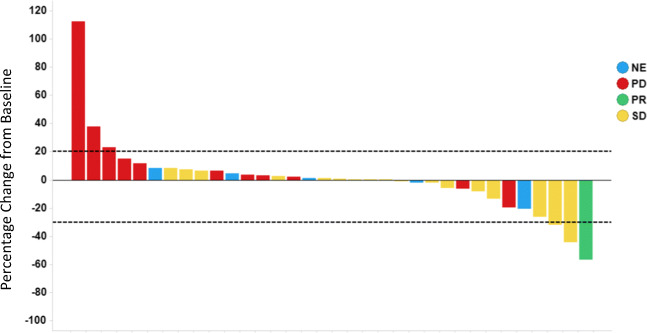
Change in tumor size at best response

Patients receiving ≥1 dose of study drug were included in the tumor response assessment modified RECIST. Thirteen patients (31%) were non-evaluable for tumor response due to missing follow-up tumor assessment. Of the 42 patients treated, one patient had a partial response lasting for 7.4 months for an objective response (ORR) of 2.4%. Three patients had unconfirmed partial responses. An additional 17 patients (41%) exhibited stable disease (SD) as their overall best response for a disease control rate (DCR) of 43%. Best change in tumor target lesion relative to baseline is presented in Fig. 1. The median progression free survival (PFS) for patients enrolled was 2.83 months (95% CI: 2.53–3.98) with a maximum of up to 10.5 months in this advanced/metastatic mesothelioma population.

### Safety

In total, 40 of the 42 (95%) patients experienced at least one adverse event possibly related to the study drug **(**Table 2**)**. Among AEs possibly related to treatment, the most frequently reported any grade AEs were fatigue (43%), nausea (43%), decreased appetite (38%), vomiting (33%), diarrhea (29%), and rash (19%) **(**Table 2**)**. The most commonly reported Grade ≥ 3 treatment-related adverse events (TRAEs) were observed for 9 patients (21%) including fatigue (10%, *n* = 4) and rash (7%, *n* = 3). Treatment-related Grade ≥ 3 hyperglycemia was reported for 2 patients (5%), including one (2%) Grade 4 event which was manageable with standard antidiabetic treatment. No Grade 5 TRAEs were reported in this cohort. With respect to treatment-related serious adverse event (SAE), fatigue (7%) was the most commonly reported, and hyperglycemia (5%) was the only Grade ≥ 3 treatment-related SAE seen in more than one patient (*n* = 2) (Supplementary Table 2).

**Table 2:** Treatment-related adverse events observed

Adverse Event (AE)	Any Grade, n (%) *N* = 42	Grade ≥ 3, n (%) *N* = 42
Subjects with ≥1 AE related to study treatment	40 (95.2)	9 (21.4)
Subjects with ≥1 SAE related to study treatment	7 (16.7)	5 (11.9)
Fatigue	18 (42.9)	4 (9.5)
Nausea	18 (42.9)	1 (2.4)
Vomiting	14 (33.3)	0
Decreased appetite	16 (38.1)	1 (2.4)
Diarrhea	12 (28.6)	0
Rash	8 (19.0)	3 (7.1)
Oral Mucositis	5 (11.9)	0
Mucosal inflammation	4 (9.5)	0
Pruritus	4 (9.5)	2 (4.8)
Blood creatinine increased	4 (9.5)	0
Weight decreased	4 (9.5)	0

*AE* adverse event, *N* total number of patients, *n* number of patients in the specified category, *SAE* serious adverse event

### Pharmacokinetic (PK) analysis

The pharmacokinetic properties of LY3023414 in patients diagnosed with mesothelioma was consistent and similar to LY3023414 pharmacokinetic properties reported in patient with other cancer type. LY3023414 pharmacokinetic is characterized by a mean apparent clearance (CL/F) and Volume of distribution (Vz/F) of 71.2 L/h and 159 L, respectively, leading to a short t1/2 (mean 1.55 h) (Table 3 and Supplementary Table 3). The supplementary Fig. 1 display graphically LY3023414 concentration time curve illustrating the similarity in LY3023414 PK profile in mesothelioma patient and in patient with other cancer type.

**Table 3:** Summary statistic of LY3023414 C_max_ and AUCτ in blood in mesothelioma patients following LY3023414 BID administration as monotherapy

		200 mg Single Dose	200 mg BID Steady State
C_max_ ng/mL	N	41	28
	GeoMean	846	846
	CV%	85	78
	90% CI	696–1027	677–1058
AUC_τ_ ng.h/mL	N	22	17
	GeoMean	2781	2597
	CV%	51	47
	90% CI	2329–3320	2149–3138

*AUCτ* area under the curve over the dose interval, *BID* twice daily (every 12 h) dosing interval, τ tau (dosing interval; 12 h for BID dosing, *Cmax* = maximum observed drug concentration, *N* number of patients, *GeoMean* geometric mean, *CV* coefficient of variation, *CI* confidence interval around the mean

### Biomarker analysis

**Fig. 2:**
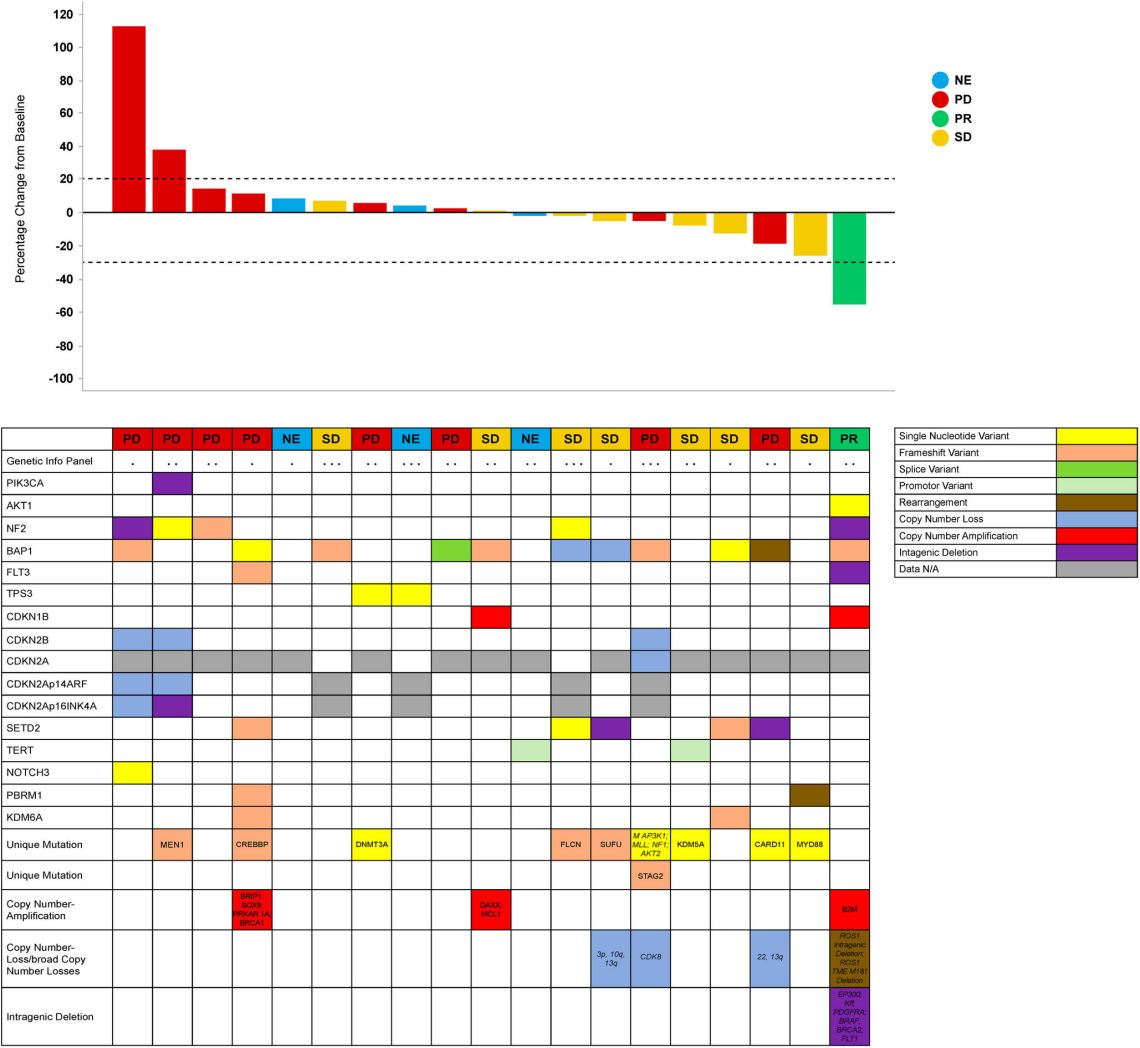
Presence of genetic alterations. # Low tumor content (approximately 20% or less); * MSK IMPACT Panel /410 Genes; ** MSK IMPACT Panel /341 Genes; *** Foundation One Panel; & showed coverage of less than 100x. unknown variant/low coverage. Note: Genetic information of tumor samples with matching tumor measurements was available for 19 patients. Unique genetic alterations were detected in some tumors (*n* = 1 each) but are not shown for the following genes: *FAT3, ZNRF3, AXIN2, INHBA, NCOR1, PTPRS, RAD51C, RYBP, SPTA1*. Since different panels were utilized not all patients had been tested for mutations in the above genes

Genetic information on tumor samples with matching tumor measurements was available for 19 patients. Consistent with previous literature, alterations of *BAP1* were identified as the most common molecular aberration, observed in a total of 11 patients, followed by *SETD2* and *NF2* alterations observed in 5 patients each **(**Fig. 2**)**. Other less common alterations involved a number of genes, including, but not limited to, *CDKN1B* and *CDKN2A/B* copy number variants. A *PIK3CA* intragenic deletion (3q26.32) was found in one patient with PD as best response and no PTEN alterations were detected. A detailed list of presence of genetic alterations is shown in Fig. 2. No obvious pattern of genetic alterations in single genes or pathways was found to be associated with anti-tumor activity. In the patient with a confirmed PR, the tumor did not harbor a *PIK3CA* mutation or PTEN loss but an alteration in the *BAP1* gene (exon 3 p.D34fs), a potent tumor suppressor implicated in PI3K signaling pathway and in the pathogenesis of malignant mesothelioma, was found. *SETD2* alterations and *CDKN1B* amplification were found in 3 and 1 SD patients, respectively.

## Discussion

Despite progress with small molecule inhibitors in many tumor types, malignant mesothelioma continues to be a challenging disease for targeted therapies and treatment options are limited. This report describes the preliminary anti-tumor activity and safety outcomes of LY3023414, a potent inhibitor of class I PI3K isoforms, mTORC1/2 and DNA-PK in patients with advanced/metastatic mesothelioma. Based on data from the phase 1a portion of the trial, the recommended phase 2 dose (RP2D) of 200 mg BID LY3023414 monotherapy showed initial signs of activity in mesothelioma patients as 2 out of 3 patients demonstrated tumor reduction [15]. In the current expansion cohort LY3023414 monotherapy showed limited activity in patients with advanced mesothelioma with 3 unconfirmed and 1 confirmed partial responses, respectively. In line with previous data, fatigue and gastrointestinal toxicities (i.e., nausea, vomiting and decreased appetite) were the most common possibly study drug-related AEs observed in this study population. These AEs were consistent in nature and frequency with the previously reported clinical safety profile for LY3023414 during dose escalation [15]. However, the AEs were largely manageable with supportive treatment or dose adjustments.

Parallel development of drug and biomarker, even in a rare disease, is feasible. In contrast to previous studies with up to 62% of PTEN loss by immunohistochemistry reported [12], only one patient evaluable for biomarkers in this mesothelioma cohort had a PTEN loss detected. Unfortunately, this patient was discontinued early and was therefore not evaluable for tumor response. Since PTEN expression may be lost by many non-genomic mechanisms, it might be necessary to determine PTEN status in tumors by both protein quantification and DNA sequencing, as neither method alone will provide comprehensive information. This could explain the different rate of PTEN loss observed in the current study. Besides *BAP1*, *NF2* alterations were among the more commonly observed. As mTOR activity is aberrantly upregulated in the case of *NF2* inactivation, suppressing mTOR activity by LY3023414 might be considered beneficial for treatment of malignant mesothelioma. However, there was no obvious association between change in tumor size and *NF2* alterations observed in this study. The patient experiencing a confirmed PR was found to harbor an intergenic deletion of *NF2* which might have contributed to the response, however, further investigation is necessary to confirm this observation. This is consistent with previous studies evaluating compounds targeting the PI3K/mTOR pathway in malignant mesothelioma. Although Apitolisib showed evidence of antitumor activity, the documented molecular changes did not correlate with the antitumor activity previously reported with PTEN loss and *PIK3CA* mutations [17]. Similarly, everolimus demonstrated limited clinical activity as a second-line therapy in patients with malignant mesothelioma [18].

Although the sample size was adequate to rule out meaningful clinical activity, due to the limited number of patients with tumor tissue available for molecular characterization and corresponding tumor assessments, no correlation can be identified from this specific cohort. Both restrict the ability to interpret the activity of LY3023414 activity in this mesothelioma patient population. However, the current data set indicate that in non-selected advanced/metastatic mesothelioma patients, there is only limited activity of LY3023414. Predictive biomarkers appear to be needed to inform further development as monotherapy.

## Conclusion

In summary, the findings of this phase 1 cohort expansion study confirm that 200 mg BID LY3023414 has an acceptable safety profile with limited single-agent activity in an unselected group of patients with advanced mesothelioma. Further studies of PI3K/mTOR inhibitors for patients diagnosed with advanced mesothelioma are warranted to identify the characteristics of patients benefitting from this class of agents as well as to elucidate potential synergistic combination therapies.

## Supplementary Information

ESM 1 (PDF 405 kb)

## Data Availability

Lilly provides access to all individual participant data collected during the trial, after anonymization, with the exception of pharmacokinetic or genetic data. Data are available to request 6 months after the indication studied has been approved in the US and EU and after primary publication acceptance, whichever is later. No expiration date of data requests is currently set once data are made available. Access is provided after a proposal has been approved by an independent review committee identified for this purpose and after receipt of a signed data sharing agreement. Data and documents, including the study protocol, statistical analysis plan, clinical study report, blank or annotated case report forms, will be provided in a secure data sharing environment for up to 2 years per proposal. For details on submitting a request, see the instructions provided at www.clinicalstudydatarequest.com.

## Declarations

### Statement of translational relevance

LY3023414 is an orally available PI3K/mTOR inhibitor, currently in development for the treatment of patients with advanced and/or metastatic cancer including malignant mesothelioma. This study evaluated safety and preliminary efficacy of LY3023414 monotherapy at the recommended phase 2 dose in patients diagnosed with advanced mesothelioma. Translational research was conducted to understand any association between genomic alteration and clinical activity observed by assessing tumor baseline samples. While limited by the number of tumor samples available, a lower than previously reported incidence of PI3K/mTOR pathway activating alterations (i.e., PTEN loss) was observed. No obvious association between change in tumor size and biomarkers assessed was identified in patients diagnosed with mesothelioma receiving LY3023414. Pharmacokinetic analysis showed similar properties reported in patients with other cancer type. LY3023414 has an acceptable safety profile with limited single-agent activity in an unselected group of patients with advanced mesothelioma.
