# Supplementary material for: Phase 1 cohort expansion study of LY3023414, a dual PI3K/mTOR inhibitor, in patients with advanced mesothelioma
Source: Invest New Drugs. 2021 Mar 4;39(4):1081–8. doi: 10.1007/s10637-021-01086-6 (PMC8280020; doi:10.1007/s10637-021-01086-6)

Supplementary Table 1. Patient Disposition

	n (%)
Enrolled	42 (100)
Treated[a]	42 (100)
**Reason for discontinuation**	
Progressive Disease	20 (47.6)
Adverse event[b]	3 (7.1)
Subject decision	5 (11.9)
Investigator decision	7 (16.7)
Death[c]	6 (14.3)

[a]Discontinuations due to AEs: dyspnea (n=1), fatigue (n=1), general disorders (n=1)

[b]1 patient discontinued due to death from AE aspiration.

Supplementary Table 2. Treatment-related Serious Adverse Events

	N=42	
**SAE related to treatment[a]**	**Any Grade, n(%)**	**Grade ≥3, n(%)**
Hyperglycemia	2(4.8)	2(4.8)
Mucosal inflammation	1(2.4)	0
Pneumonitis	1(2.4)	0
Cardiac failure	1(2.4)	1 (2.4)
Fatigue	3(7.1)	1(2.4)
Dyspnoea	2(4.8)	1(2.4)

[a]Patients may be counted in more than one category

Abbreviation: SAEs=serious adverse events

Supplementary Table 3. Summary Statistics of LY3023414 Disposition Pharmacokinetic Parameters Following Single LY3023414 Monotherapy Dose in Patients with Mesothelioma

LY3023414 Dose mg		t1/2 (h)	CL/F (L/h)	V_z/F (L)
	N	22	22	22
	GeoMean (CV%)	1.55 (29)	71.2 (51)	159 (61)
	90% CI	(1.39-1.72)	(59.6-84.9)	(129-195)
	Range	1.1 – 2.6		

Abbreviations: t½= terminal half-life; CL/F = apparent clearance following extravascular oral administration; Vz/F = apparent volume of distribution; N = number of patients; GeoMean = geometric mean, CV = coefficient of variation; CI = confidence interval around the mean.

Supplementary Figure 1. LY3023414 Concentration Versus Time Curve (mean+/-SD) in Patients with Mesothelioma after Single Dose (top panel) and Multiple Doses (lower panel) Administration of LY3023414 200 mg BID Monotherapy Administration

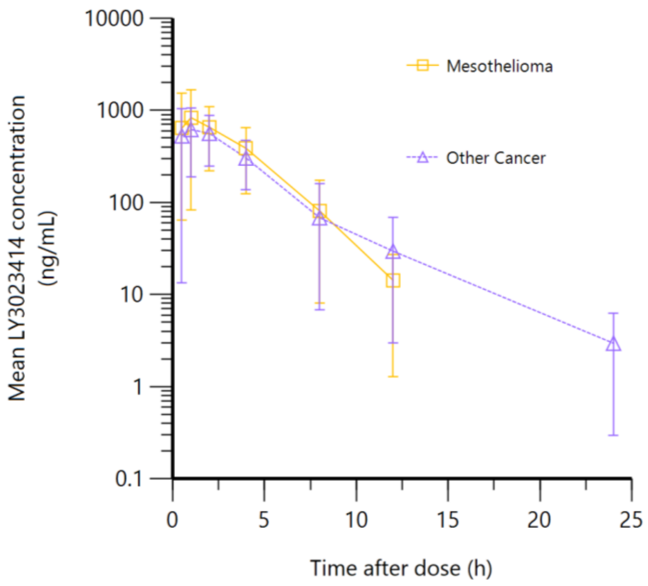

Supplement: Supplementary file 1 — (PDF 405 kb) [file 10637_2021_1086_MOESM1_ESM.pdf]
